# Comparison of the effect of oral care with four different antiseptics to prevent ventilator-associated pneumonia in adults: protocol for a network meta-analysis

**DOI:** 10.1186/s13643-017-0496-5

**Published:** 2017-05-19

**Authors:** Zhigang Zhang, Yuying Hou, Jun Zhang, Bo Wang, Juxia Zhang, Ailing Yang, Ge Li, Jinhui Tian

**Affiliations:** 1 Department of Intensive Care Unit, The First Hospital of Lanzhou University, Lanzhou, China; 2 The First Clinical Medical College of Lanzhou University, Lanzhou, China; 3 School of Nursing, Gansu University of Traditional Chinese Medicine, Lanzhou, China; 4 Department of Nursing, Rehabilitation Hospital of Gansu Province, Lanzhou, China; 5 Department of Nursing, Gansu Provincial Hospital, Lanzhou, China; 6 Department of Nursing, Lanzhou University Second Hospital, Lanzhou, China; 7 School of Public Health, Tianjin University of Traditional Chinese Medicine, Tianjin, China; 8 Evidence-Based Medicine Center, School of Basic Medical Sciences, Lanzhou University, Lanzhou, China; 9 Key Laboratory of Evidence Based Medicine and Knowledge Translation of Gansu Province, No. 199, Dong gang West Road, Chengguan District, Lanzhou, Gansu China

**Keywords:** Ventilator-associated pneumonia, Oral care, Antiseptic, Network meta-analysis

## Abstract

**Background:**

Ventilator-associated pneumonia (VAP) is defined as the occurrence of pneumonia in patients receiving mechanical ventilation for more than 48 h after endotracheal intubation. The implementation of effective oral care with antiseptics may reduce the incidence of ventilator-associated pneumonia. However, previous studies have been unclear about the best antiseptic for this purpose. Therefore, present protocol proposed to perform a network meta-analysis to evaluate the efficacy of different antiseptics to prevent ventilator-associated pneumonia.

**Methods/design:**

We will search CNKI, WanFang database, PubMed, Web of Science, Cochrane Library, EMBASE, SinoMed from their inception to March 2016. There are no restrictions on language, publication year, or publication type. Only randomized clinical trials (RCTs) with antiseptics to prevent ventilator-associated pneumonia will be considered. Study selection and data collection will be independently performed by two reviewers. Risk of bias assessments will be completed using the Cochrane risk of bias scale. The primary outcome is VAP morbidity. A network meta-analysis will be conducted to compare the effect of four different antiseptics on patient-relevant efficacy. Subgroup analyses will be performed by the type of setting and length of mechanical ventilation, and sensitivity analyses will be conducted to assess the robustness of the findings.

**Discussion:**

Oral care to prevent ventilator-associated pneumonia has been widely used. The efficacy of usual oral antiseptics have been assessed mainly using traditional meta-analysis. However, it was unclear which oral care solution is best used for oral care and there were no head-to-head RCT to compare the efficacy of four antiseptics. The proposed network meta-analysis will compare four antiseptics and rank the results using network meta-analysis to decide which was the best.

**Systematic review registration:**

PROSPERO CRD42016038088

**Electronic supplementary material:**

The online version of this article (doi:10.1186/s13643-017-0496-5) contains supplementary material, which is available to authorized users.

## Background

Ventilator-associated pneumonia (VAP) is a form of hospital-acquired pneumonia (HAP) and is defined as the occurrence of pneumonia in patients receiving mechanical ventilation for more than 48 h after endotracheal intubation [1]. VAP remains the leading cause of nosocomial infection in intensive care units (ICU), affecting 10–30% of patients receiving mechanical ventilation. VAP is a common complication of intubation and causes lengthening of hospital stay with increased use of healthcare resources costing nearly US$50,000–US$57,000 per occurrence [2] and increasing the risk of mortality to 15–45% [3]. As such, prevention of VAP is a key component of improving ICU care.

Numerous factors have been associated with VAP, such as aspiration, reintubation, supine positioning, and failed subglottic aspiration. Several studies strongly support the hypothesis that the most important mechanism for the development of VAP is the aspiration of colonized oropharyngeal secretions into the lower respiratory tract [4]. Oral bacterial colonization results from an accumulation of debris in the oral cavity. Adequate salivary flow is an important factor for the maintenance of oral health through its antimicrobial, lubricating, and buffering properties [5]. However, as mechanically ventilated patients cannot be fed orally, their salivary secretions decrease, and there is a marked reduction in self-cleansing of the oral cavity. Additionally, an endotracheal tube can hamper the oral cavity and limit access for oral care, causing a pH imbalance in the oral cavity, decreasing saliva production, and making it easier for bacteria to colonize [6].

Many recommendations have been made and applied in clinical practice to reduce VAP, such as sedation protocols with more interactive patients, daily interruption of sedation, maintenance of a semi-recumbent position (30° to 45°), and maintenance of oral care [7]. In all of these, oral care is considered crucial for preventing pneumonia [8]. In several previous studies [6, 9–12], the use of *chlorhexidine* for oral care has proven to be effective in the prevention of VAP. The Centers for Disease Control and Prevention (CDC) recommends oral hygiene with chlorhexidine in patients in the perioperative period of cardiac surgery. However, such recommendations have not been made for other patient groups. Additionally, previous meta-analyses assessing the effect of oral antiseptics on rates of ventilator-associated pneumonia had different scopes. A meta-analysis by Sonia et al. [5] found that the use of 2% chlorhexidine had a beneficial effect on the prevention of VAP, especially in cardiosurgical patients. Another meta-analysis by Berry et al. [13] showed that the effectiveness of *sodium bicarbonate* was unclear. In addition, Hadi’s study [14] found that a herbal oral mouth wash consisting of persica and matrica had an effect on *Streptococcus pneumoniae* and *Staphylococcus aureus* in the oropharynx of mechanically ventilated patients.

Previous meta-analyses have not focused on any head-to-head comparisons of different antiseptics [15]. However, decision-makers and clinical care workers need to know the rankings of a set of alternative options and not only whether one option is better than another. Network meta-analyses allow a unified, coherent analysis of all RCTs that compare oral care with antiseptics in VAP patients, while fully respecting randomization within the included trials. In addition, such a meta-analysis allows the ranking of antiseptics by efficacy and safety by taking advantage of the measured differences versus common comparators, even if the antiseptics are not or only insufficiently compared head to head [16, 17].

For these reasons, the primary objective of this study is to carry out a network meta-analysis comparing the efficacy and safety of oral care with different antiseptics for the prevention of VAP in adults based on existing RCTs and ranking these antiseptics based on their performance.

## Methods and analysis

### Protocol and registration

A standard protocol will be followed for all review steps. Our protocol has been registered on PROSPERO (CRD42016038088), and our manuscript will conform to “The Preferred Reporting Items for Systematic Reviews and Meta-Analyses Protocols (PRISMA-P)” guidelines [18]. A completed PRISMA-P recommendation checklist is included as an additional file (see Additional file 1).

### Data sources and searches

Eligible studies will be acquired through a systematic database search of PubMed, Web of Science, Cochrane Library, EMBASE, and SinoMed from their inception to March 2016. In addition, we will also search conference abstracts from the Society of Critical Care Medicine, the European Society for Intensive Care Medicine, the American Thoracic Society, and the American College of Chest Physicians, as well as the Clinicaltrials.gov and Controlled-trials.com along with the bibliographies of eligible studies and relevant review articles or meta-analyses and the reference lists of identified studies. Using the PubMed database as an example, we will use the following strategy: (pneumonia OR (ventilator associated pneumonia) OR (hospital acquired pneumonia) OR (nosocomial pneumonia) OR HAP OR VAP) AND (oral care OR (oral hygiene) OR (topical antiseptics)) (an additional file shows this in more detail (see Additional file 2).

A highly sensitive strategy developed by Cochrane Collaboration for identifying RCTs in MEDLINE, known as sensitivity maximizing version, will be used [19].

### Study eligibility criteria

Articles that meet the following criteria will be included.

#### Inclusion of participants

Hospitalized adult patients (>18 years) who have a device to continuously assist or control respiration through a tracheostomy will be included. We will exclude studies with patients that had not undergone mechanical ventilation for more than 48 h prior to enrolment.

#### Interventions

Studies comparing one antiseptic regimen with a placebo or comparison antibiotic regimen will be included, as will trials evaluating monotherapy versus combination therapy. We will classify antiseptics into groups as follows:

1. Chlorhexidine
2. Povidone-iodine
3. Five percent sodium bicarbonate
4. Herbal mouthwash of Matrica(R) (chamomile extracts) 10%

The above list is not exhaustive. If pharmacological interventions are identified that we are not currently aware of, we will consider them as eligible and include them in the network analysis if they are used primarily for the prevention of VAP.

#### Outcome

The primary outcome of this study is VAP morbidity. Secondary outcomes include the length of intensive care stay, the length of hospital stay, and the duration of mechanical ventilation.

#### Types of study

We will consider RCTs for this network meta-analysis irrespective of language, publication status, or date of publication. The RCT included in this study is defined as a RCT that explicitly illustrates the random sequence generation method. We will exclude studies of other designs because of the risk of bias.

#### Study selection

Studies will be selected according to the methods described in the Cochrane Handbook for Systematic Reviews of Interventions [19]. The title, abstract, and keywords of every retrieved record will be scanned independently by two reviewers to determine which studies require further assessment. After excluding the duplicate and unrelated studies, the full article will be obtained and examined in detail to determine whether the study fulfilled the eligibility criteria. If there is any doubt about a study, a consensus will be used to resolve the issue and a third and fourth author will be consulted if required. Study selection will be documented using the PRISMA extension statement for the reporting of systematic reviews incorporating network meta-analyses (PRISMA-NMA) [20].

### Data extraction

Two authors will extract data for our network meta-analysis from the included RCTs. The following data will be extracted using standardized data extraction forms: first author name, publication year, journal, country, patient characteristics (number of patients, patients’ baseline, interventions, control treatment), outcome measures (VAP morbidity, the length of intensive care stay, duration of mechanical ventilation), institution, and sponsor. Any disagreements will be resolved by a third reviewer. If necessary, we will try to contact the corresponding authors for more information.

### Assessment of risk of bias in included studies

The Cochrane risk of bias tool will be adopted to assess the risk of bias for each RCT. Specifically, we will assess the risk of bias in the included trials for the following domains using the methods below: allocation sequence generation, allocation concealment, blinding of participants and personnel, blinding of outcome assessment, incomplete outcome data, selective reporting bias, and other biases. After the independent quality assessment, two authors will discuss each article with regard to the assessment level, and if need be, with another author. Each potential source will be graded as high, low, or unclear level of bias and provide a quote from the study report together with a justification for our judgment in the “Risk of bias” table.

### Assessment of publication bias

A funnel plot of sample and effect size will be constructed to investigate the presence of publication bias. If publication bias is suspected, i.e., significant asymmetry is found after a visual inspection of the funnel plot, we will include a statement in our results and our summary of findings table, with a corresponding note of caution in our discussion.

### Statistical analysis

A network meta-analysis will be performed to assess the relative outcomes of different antiseptics. We will perform this analysis using the WinBUGS V1.4.3 software package for multiarm trials [20]. Other analyses will be performed using the Stata13.0 software packages [15]. We will calculate odds ratios (OR) with its 95% CIs for dichotomous data and mean differences (MD) with its 95% CIs for continuous data. Weighted mean differences will be used for data measured on the same scale and for which the same units are used; otherwise, standardized mean differences will be used (http://www.cochrane.org/handbook).

Bayesian methods will be used under both fixed-effect and random-effects multiple treatment comparisons (MTC) for indirect comparisons [21]. The fixed-effect model assumes no variance between studies, while the random-effects model assumes homogeneous variance between studies. Posterior estimates will be derived for Bayesian methods, using Gibbs sampling via Markov chain Monte Carlo simulation in WinBUGS V1.4.3. In order to control for random error due to the choice of priors, the network meta-analyses will be performed for three different priors as recommended by the NICE DSU [22]. We will use a normal distribution with large variance (10,000) for treatment effects. The priors that we will use in the three different chains will be:

- Chain 1: treatment effect: (*d*(*k*) = 0); mu(*i*) = 0;
- Chain 2: treatment effect: (*d*(*k*) = −1); mu(*i*) = −3;
- Chain 3: treatment effect: (*d*(*k*) = 2); mu(*i*) = random

Between −7 and +7 (based on Excel generated random numbers); where *d*(*k*) = treatment effect of experimental intervention “*k*” compared with reference and mu (*i*) = treatment effect of the experimental intervention when compared with the control in the trial “*i.*” If the three different priors produce similar results for the meta-analysis, that is, the models converge, then, we will consider the results reliable [23].

The Brooks-Gelman-Rubin method will be used to assess convergence. Using this process, a potential scale reduction factor (PSRF) is calculated by comparing within-chain and between-chain variance. A PSRF very close to 1 is considered to indicate an approximate convergence.

The cumulative probability of the treatment ranks (i.e., the probability that the treatment is within the top two, the probability that the treatment is within the top three, etc.) will be presented in graphical form (Surface Under the Cumulative Ranking curve (SUCRA)) [24]. SUCRA values are expressed as percentages; if an intervention is clearly the best, its SUCRA value would be 100%, and if an intervention is clearly the worst, its SUCRA value would be 0%. If all interventions are equal, all SUCRA values would be expected to be near 50%. We will also plot the probability that each treatment is best for each outcome (rankograms), which is generally considered more informative [24, 25].

### Assessment of heterogeneity

We will assess statistical heterogeneity by comparing the model fit between the fixed-effect model of the meta-analysis and random-effects model of the meta-analysis. The *I* ^2^ statistic will be used to measure heterogeneity among the trials in each analysis, with results in a 0–100% range quantifying the proportion of variation in the effect, which is due to inter-study variation. We will consider an *I* ^2^ value of 75 to 90% as substantial heterogeneity.

### Assessment of inconsistency

An inconsistency model and a consistency model will be fitted to the data. Model selection will be based on deviance information criteria (DIC), with a difference of five points suggesting an important difference. Next, a *Z* test will be performed to examine the inconsistency of the model. If a loop exists in the network (e.g., A-B-C), each comparison in this loop (e.g., A vs C) may have confer an indirect value derived from other comparisons in the loop (e.g., A vs B and B vs C), and this indirect value will be compared with its direct value. Then, the *Z* value and its corresponding *P* value will be calculated, and if the *P* value is >0.05, no statistically significant difference will be noted.

### Subgroup analysis

Several subgroup analyses will be performed if applicable for the following:

1. Studies in different settings
2. Length of mechanical ventilation

We will use the formal test for subgroup interactions in RevMan 2014.

### Sensitivity analysis

To assess the robustness of the pooled results and to explore possible reasons for heterogeneity, we will undertake sensitivity analysis based on random sequence generation and allocation concealment of methodological quality, comparing studies with a low risk of bias to those with high or unclear risk of bias.

## Discussion

Oral care to prevent ventilator-associated pneumonia has been widely used. The efficacy of usual oral antiseptics have been assessed mainly using traditional meta-analysis. However, it was limited and difficult currently to assess more than two inventions by traditional meta-analysis. It was unclear which oral care solution is best used for oral care, and there were no head-to-head RCT to compare the efficacy of four antiseptics. The proposed network meta-analysis will compare four antiseptics and rank the results using network meta-analysis to decide which was the best for the clinical workers to provide reliable evidence and better promote patient health.

### Ethics and dissemination

No primary data will be collected in this study, and as such, no additional formal ethical assessment or informed consent are required. Our review will provide relative estimates of effectiveness of each antiseptic and will evaluate the quality of the evidence in a thorough and consistent manner using the GRADE approach [26]. The results will be published in a peer-reviewed journal and disseminated both electronically and in print.

## Additional files

Additional file 1: PRISMA-P (Preferred Reporting Items for Systematic Review and Meta-Analysis Protocols) 2015 checklist: recommended items to address in a systematic review protocol. (PDF 172 kb)

Additional file 2: PubMed search strategy. (PDF 53 kb)

## Abbreviations

CDC: Centers for Disease Control and Prevention
HAP: Hospital-acquired pneumonia
ICU: Intensive care units
MTC: Multiple treatment comparisons
RCTs: Randomized clinical trials
VAP: Ventilator-associated pneumonia
