# Supplementary material for: Comparison of the effect of oral care with four different antiseptics to prevent ventilator-associated pneumonia in adults: protocol for a network meta-analysis
Source: Syst Rev. 2017 May 19;6:103. doi: 10.1186/s13643-017-0496-5 (PMC5437639; doi:10.1186/s13643-017-0496-5)
Supplement: Supplementary file 2 — PubMed search strategy. (PDF 53 kb) [file 13643_2017_496_MOESM2_ESM.pdf]

Additional file

Additional file 2: Serch strategy of PubMed

#1 "Pneumonia, Ventilator-Associated"[Mesh]

#2 "Ventilator associated pneumonia"[Title/Abstract]

#3 VAP[Title/Abstract]

#4 "hospital acquired pneumonia"[Title/Abstract]

#5 HAP[Title/Abstract]

#6 nosocomial pneumonia[Title/Abstract]

#7 pneumonia[Title/Abstract]

#8 #1 OR #2 OR #3 OR #4 OR #5 OR #6 OR #7

#9 "Oral Hygiene"[Mesh]

#10 "Oral Hygiene"[Title/Abstract]

#11 "oral care"[Title/Abstract]

#12 "topical antiseptics"[Title/Abstract]

#13 #9 OR #10 OR #11 OR #12

#14 "Randomized Controlled Trial" [Publication Type]

#15 "Randomized Controlled Trial"[All Fields]

#16 "Random"[All Fields]

#17 #14 OR #15 OR #16

#18 #8 AND #13 AND #17
